# Inflammatory Markers in Obsessive-Compulsive Disorder: A Systematic Review and Meta-Analysis

**DOI:** 10.1192/j.eurpsy.2022.753

**Published:** 2022-09-01

**Authors:** M. Constable, R. Hou

**Affiliations:** 1 University of Southampton, Faculty Of Medicine, Southampton, United Kingdom; 2 University of Southampton, Department Of Psychiatry, Southampton, United Kingdom

**Keywords:** inflammatory markers, systematic review, psychiatry, obsessive-compulsive disorder

## Abstract

**Introduction:**

A growing number of studies have examined the link between inflammatory markers (IM) and the pathophysiology of obsessive-compulsive disorder (OCD). However, this association has yet to be fully identified.

**Objectives:**

This review aims to systematically evaluate evidence from studies examining peripheral IM in adult participants with OCD compared to controls. IM included: CRP, TNFa, IFNγ, IL1/4/6/10.

**Methods:**

Databases used for literature searching: Medline, Embase, PsycINFO (until October 2021). Studies that examined IM in the blood of adult OCD and control groups were included. Screening and data extraction adhered to PRISMA guideline standards. The quality assessment utilised funnel plots and the approach developed by Hawker et al. 2002. A random-effects meta-analysis model was adopted. PROSPERO reference number: CRD42021284766.

**Results:**

The systematic review (19 studies, 1,225 participants) and meta-analysis (12 studies, 796 participants) had an average quality assessment score of 28.3 (medium quality) and 30.7 (high quality), respectively. The average heterogeneity of each IM analysed was 76.6%. Totalled, each study and IM analysis showed more insignificant differences (n=35) than significant differences (n=25). The meta-analysis revealed no significant difference for overall IM assessments. However, a sub-analysis of IL6 (excluding studies using serum or lipopolysaccharide stimulation) found significantly lower levels of IL6 within the OCD group (effect size: 3.98 and 95% CI: 0.43,7.53).

**Conclusions:**

This is an up-to-date systematic review examining IM in OCD. Insignificant results found may have resulted from the relatively high heterogeneity or varied study designs. One sub-analysis of IL6 identified an association, although further studies are required with larger sample sizes and fewer disparities.

**Disclosure:**

No significant relationships.

